# Integrative analysis of multi-omics data for discovery of ferroptosis-related gene signature predicting immune activity in neuroblastoma

**DOI:** 10.3389/fphar.2023.1162563

**Published:** 2023-07-13

**Authors:** Jiajian Hu, Fengju Song, Wenjuan Kang, Fantong Xia, Zi’an Song, Yangyang Wang, Jie Li, Qiang Zhao

**Affiliations:** ^1^ Tianjin Key Laboratory of Cancer Prevention and Therapy, Department of Pediatric Oncology, National Clinical Research Center for Cancer, Tianjin’s Clinical Research Center for Cancer, Tianjin Medical University Cancer Institute and Hospital, Tianjin, China; ^2^ Key Laboratory of Molecular Cancer Epidemiology, Department of Epidemiology and Biostatistics, National Clinical Research Center of Cancer, Tianjin Medical University Cancer Institute and Hospital, Tianjin, China

**Keywords:** ferroptosis, survival, gene, immune, neuroblastoma

## Abstract

Immunotherapy for neuroblastoma remains unsatisfactory due to heterogeneity and weak immunogenicity. Exploring powerful signatures for the evaluation of immunotherapy outcomes remain the primary purpose. We constructed a ferroptosis-related gene (FRG) signature by least absolute shrinkage and selection operator and Cox regression, identified 10 independent prognostic FRGs in a training cohort (GSE62564), and then verified them in an external validation cohort (TCGA). Associated with clinical factors, the signature accurately predicts overall survival of 3, 5, and 10 years. An independent prognostic nomogram, which included FRG risk, age, stage of the International Neuroblastoma Staging System, and an MYCN status, was constructed. The area under the curves showed satisfactory prognostic predicting performance. Through bulk RNA-seq and proteomics data, we revealed the relationship between hub genes and the key onco-promoter MYCN gene and then validated the results in MYCN-amplified and MYCN–non-amplified cell lines with qRT-PCR. The FRG signature significantly divided patients into high- and low-risk groups, and the differentially expressed genes between the two groups were enriched in immune actions, autophagy, and carcinogenesis behaviors. The low-risk group embodied higher positive immune component infiltration and a higher expression of immune checkpoints with a more favorable immune cytolytic activity (CYT). We verified the predictive power of this signature with data from melanoma patients undergoing immunotherapy, and the predictive power was satisfactory. Gene mutations were closely related to the signature and prognosis. AURKA and PRKAA2 were revealed to be nodal hub FRGs in the signature, and both were shown to have significantly different expressions between the INSS stage IV and other stages after immunohistochemical validation. With single-cell RNA-seq analysis, we found that genes related to T cells were enriched in TNFA signaling and interferon-γ hallmark. In conclusion, we constructed a ferroptosis-related gene signature that can predict the outcomes and work in evaluating the effects of immunotherapy.

## Introduction

Neuroblastoma (NB) is the most common extracranial solid malignancy in children (London et al., 2005). Accounting for 15% of cancer-related mortalities, it has the characteristics of rapid metastasis and strong malignancy (Ishola and Chung, 2007). The prognosis of patients is affected by factors such as age, stage, histology, MYCN status, and DNA ploidy. A significant amount of effort has been directed toward the improvement of treatment results in advanced neuroblastoma; however, the prognosis of about half of the patients remains poor (Hara, 2012).

To overcome treatment difficulties, additional targeted therapies for adult malignancies have been tried for NB, yet powerful treatments are still being explored (Louis and Shohet, 2015; Whittle et al., 2017; Lochmann et al., 2018). NB denotes a variable biological character. Although an extensive description of copy number alterations are found in NB, few single gene alterations have been shown to be driver mutations in NB oncogenesis (Combaret et al., 2015). Therefore, the discovery of specific drugs remains one difficult obstacle (Schulte et al., 2013; Moreno et al., 2020). Besides, NB can spontaneously downregulate MHC-I and inhibit antitumor immune components in the microenvironment (Wienke et al., 2021). Stimulating the immunogenicity of NB cells and transforming “cold tumors” into “hot tumors” has gradually become a new research hot spot. There is an urgent requirement for more effective biomarkers to guide the immune reaction such as in immune checkpoint blockage therapy (Whittle et al., 2017).

Ferroptosis is recognized as an iron-dependent and reactive oxygen species (ROS)–dependent cell death, which may act as an adaptive process to be critical for eradicating carcinogenic cells (Xie et al., 2016; Dixon, 2017). Besides, ferroptosis is closely related to other death modes like autophagy and may play a vital role in shaping the tumor immune microenvironment (Dai et al., 2020). Several studies have applied ferroptosis-related genes (FRGs) to predict prognosis and evaluate components of the immune microenvironment of various malignancies, although not yet in NB (Liu et al., 2020). However, ferroptosis may play an important role in the occurrence and development of ferroptosis, and there might be a variety of therapeutic targets in the ferroptosis pathway of NB (Geng et al., 2018; Monteleone et al., 2021). Therefore, the interplay between ferroptosis and immunity in NB has to be elucidated, as does the identification of the key prognostic FRGs that regulate the microenvironment.

In the present study, we first shed light on the role of ferroptosis-related genes in immunity, metabolism, and autophagy of NB. Furthermore, we developed and externally validated a ferroptosis-related signature that can accurately predict the 3-, 5-, 10-year prognosis of patients. By analyzing the RNA-seq of tissues, cell lines, proteomics, and qRT-PCR *in vitro*, we explored the relationship between hub genes and the key onco-promoter MYCN. The RiskScore calculated by the signature can to a certain extent divide the disease into “hot tumor” and “cold tumor,” besides reflecting the mutation load of tumors. The nodal genes AURKA and PRKAA2 were correlated with prognosis and verified by immunohistochemistry (IHC) to be related to the degree of malignancy. Moreover, we found that genes related to T cells were enriched in TNFA signaling and interferon-γ hallmark via single-cell RNA-seq analysis. Finally, this signature had proved meaningful to understand the immune microenvironment and guide immune checkpoint blockade.

## Materials and methods

### Data collection and preprocessing

In this study, the RNA-seq data and corresponding clinical information of 498 NB samples, which were downloaded from the Gene Expression Omnibus (GEO, http://ncbi.nlm.nih.gov/geo)—GSE62564 database, were named the training set (Su et al., 2014). While the validation cohorts were obtained from The Cancer Genome Atlas (TCGA) (121 NB) (https://portal.gdc.cancer.gov/) (Wang et al., 2016). We performed transcriptomic and immune profiling on tumor biopsies from melanoma patients treated with combined anti-PD-1 and anti-CTLA-4 (n = 51) to find the correlates of responder (n = 35) vs. non-responder (n = 16) to the therapy from Tumor Immune Dysfunction and Exclusion (TIDE) (http://tide.dfci.harvard.edu/download/) (Gide et al., 2019). The proteomics data of 49 patients with NB were obtained from the literature (Birte et al., 2021) We obtained expression profiling in NB cell lines (29 cell lines and 38 samples) from the GSE19274 database (Cole et al., 2011). After removing data with unknown MYCN status, we retained 21 MYCN-amplified and 13 MYCN–non-amplified cell lines. A total of 259 FRGs were acquired from the FerrDb data set (http://www.zhounan.org/ferrdb/). The data on RNA-seq were log2 transformed. In terms of the proteomics data, we used the Perseus software (2.0.6.0) to preprocess the data as follows: contaminants and protein groups identified by a single peptide were filtered from the data set. The proteinGroup LFQ intensities were log2 transformed to reduce the effect of outliers. The missing values were replaced from the normal distribution. We used “limma,” “ggplot2,” “ggpubr,” and “ggstatsplot” packages to verify the correlation between molecule expression and MYCN status.

### Construction and validation of the prognostic ferroptosis-related risk signature

To construct an FRG signature, we proceed as follows: 1. in total, 236 FRG mRNAs were obtained by intersecting between mRNAs of the training set and the FRGs, 2. FRGs with prognosis were evaluated by univariate Cox regression analysis, 3. the DEGs with a *p* < 0.01 were chosen as the candidate variables and entered into the least absolute shrinkage and selection operator (LASSO) regression, 4. the stepwise multivariate Cox regression analysis was subsequently applied for reserved genes from the LASSO regression analysis to select the candidate DEGs tested by the Akaike information criterion (AIC) for identifying the risk signature, 5. model: RiskScore = $\sum_{i=1}^{n}EXPi\times Coei$ , where EXP and Coe represent the expression value and regression coefficient of the DEGs from the multivariate cox regression analysis.

Then, patients with their corresponding calculated RiskScore were divided into the low- and high-risk prognostic groups based on the median risk value. Subsequently, the Kaplan–Meier survival curves and receiver operating characteristic (ROC) curve were performed to test the prognostic and predictive efficacy of the FRG risk signature. Additionally, the prognostic value derived from the training set was then applied to the validation set (TCGA), the immunotherapy data set of melanoma, and the NB cell data set (GSE19274) to calculate the RiskScores.

### Survival analysis of NB FRG signature and correlations with pathological features

A nomogram was established through the univariate and multivariate Cox regression analysis, employing the independent prognostic factors in the training set. The C-index and ROC analysis of the training and validation sets were used to value the availability of the nomogram.

### Functional enrichment analysis

Based on the computational algorithm of the gene set enrichment analysis (GSEA) for analyzing the molecular profiles of the data set, we compared the low- and high-risk groups from the training cohort to identify the enriched pathway. The Gene Set Variation analysis (GSVA) was used to detect the difference in expression with the RiskScore. Meanwhile, the c2.cp.v7.4.symbols.gmt gene sets were downloaded from the Molecular Signatures Database (http://www.Gsea-msigdb.org) and then calculated by using single-sample gene set enrichment analysis (ssGSEA) in the “GSVA” R package (Hanzelmann et al., 2013). Furthermore, the DEGs between the high- and low-risk groups were identified by |log2FC|>2 and adjusted *p* < 0.05, and then functionally annotated by the Gene Ontology (GO).

### Evaluation of the immune landscape

The penetration fraction was calculated using the ssGSEA for 28 immune cells. The immune scores and stromal scores of NB patients were calculated using the “estimate” package (ESTIMATE algorithm: estimation of stromal and immune cells in malignant tumor tissues using expression data) (Yoshihara et al., 2013). As in a previous study, there were seven steps involved in the activation of anti-cancer immunity cycle, and these steps could be downloaded from the tracking the tumor immunophenotype web (http://biocc.hrbmu.edu.cn/TIP/index.jsp) (Liwen et al., 2018) and scored by using the ssGSEA based on the gene expression of each sample. In addition, CYT, which reflects the cell killing function by a geometric mean of gene expressions of granzyme A (GZMA) and perforin 1 (PRF1), could be put to value immune-mediated attack against cancer cells (Rooney et al., 2015). Moreover, a Wilcoxon rank-sum test was performed to examine the association between the signature group and immune checkpoints, which included PD-L1, PD-1, CTLA-4, and IDO-1. The univariate Cox regression and Kaplan–Meier survival analysis on each immune checkpoint were performed.

### RiskScore of FRG signature correlates with genome instability and tumor mutation burden

We downloaded the somatic mutation data of 209 NB patients from the TCGA database and calculated the TMB for each case using the formula. Then, we used the “maftools” package to visualize the mutational profiles (Mayakonda et al., 2018). Toward the end, we performed a correlation analysis between the TMB and RiskScore.

### Cell culture and quantitative real-time polymerase chain reaction

Human NB cell lines, SK-N-AS (MYCN–non-amplified) and SK-N-BE2 (MYCN-amplified), were purchased from the American Type Culture Collection (ATCC, United States). The cell lines were cultured in DMEM (VivaCell, China), enriched with 10% FBS (VivaCell, China), and maintained in a humidified incubator at 37°C, 5% CO2. The FRGs with their consistent correlation with MYCN expression between both tissue and cell databases were included for the qRT-PCR analysis.

The total RNA of each cell line was extracted via TRIzol (Thermo, United States), followed by reverse transcription into cDNA with PrimeScript™ RT Master Mix (TaKaRa, Japan) according to the manufacturer’s instructions. qPCR was carried out using the TB Green^®^ Premix Ex Taq^™^ II kit (TaKaRa, Japan). The amplification reaction for cDNA detection was carried out for 40 cycles. Each cycle contained denaturation at 95°C for 30 s, annealing for 5 s, and an extension at 60°C for 20 s. β-actin served as the internal control. The relative expression levels were quantified with the 2^−ΔΔCt^ method. The primer sequences are listed in Supplementary Table S1.

### Screening nodal genes and immunohistochemistry analysis

To screen out the nodal gene in this FRG signature, we used the integrated interactions database (http://iid.ophid.utoronto.ca/search_by_proteins/), which can construct tissue-specific protein–protein interaction (PPI) networks across species. The two selected genes were analyzed for overall survival (OS) and event-free survival (EFS). A total of 10 NB tissues were obtained from the Tianjin Medical University Cancer Institute and Hospital, which included INSS stage IV and other stages (INSS I–III, IVS). Our study was approved by the Ethics Committee of the Tianjin Medical University Cancer Institute and Hospital. A written informed consent was signed by every patient or legal guardian before the study started. Primary antibodies that included PRKAA2 (18167-1-AP, 1:100 for IHC) purchased from Proteintech and AURKA (DF6845, 1:50 for IHC) purchased from Affinity were applied. IHC was performed according to previously described procedures (Tian et al., 2021). The IHC score was calculated with staining percentage and intensity (Yin et al., 2021). Two experienced pathologists were blinded to the clinical information and independently assessed the slides.

### Single-cell RNA sequence analysis

We obtained single-cell RNA-seq profiles (NB02, NB16, NB23, and NB24) from GSE147766 (Verhoeven et al., 2022). Using the “Seurat” and “SingleR” packages to conduct data analysis, we moved cells with a number of features <50 and genes detected <3 cells. By subjecting the 1,500 feature genes to the principal component analysis (PCA), we obtained single-cell clusters. The “SingleR” was used for cell-type annotation. Then, we recognized the marker genes of each cell type by absolute log2 fold change >0.5 and an adjusted *p* <0.05. Expression correlation assays between the RiskScore and T-cell marker genes were performed using the Spearman’s coefficient correlation on the GSE62564 and TCGA data sets, respectively. The GSEA hallmark pathways enriched in the intersection genes above, ordered by the −log10 false discovery rate (FDR).

### Statistical analysis

All statistical analyses were performed using R (version 4.0.3) and its appropriate packages (Sepulveda, 2020). The statistical significance was defined with a two-tailed *p* < 0.05. We used either Pearson’s r correlation or Spearman’s rank-order correlation to measure the correlation between two continuous variables. The 3-, 5-, and 10-year prognosis were taken as follow-up nodes (Hassanzadeh et al., 2017; Tuomainen et al., 2020; Berberi, 2021). The comparison of a continuous variable in two or more than two groups was made using either a parametric test (Student’s *t*-test or analysis of variance) or non-parametric test (Wilcoxon rank-sum test or Kruskal–Wallis test) if the variable was normally distributed.

## Results

### Construction and validity of prognostic gene signature related to NB and ferroptosis

**FIGURE 1:**
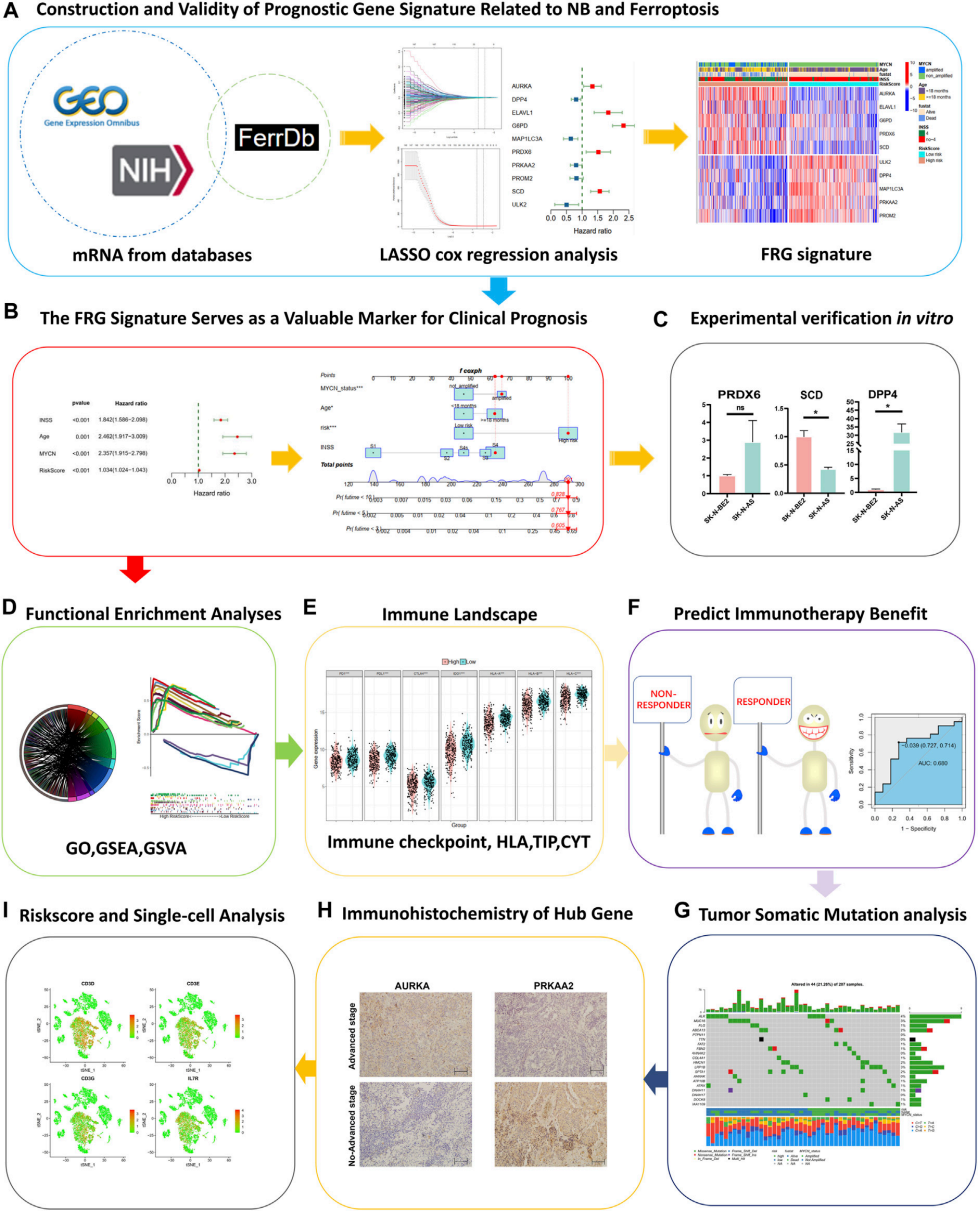
Study design and workflow of the present study.

**FIGURE 2:**
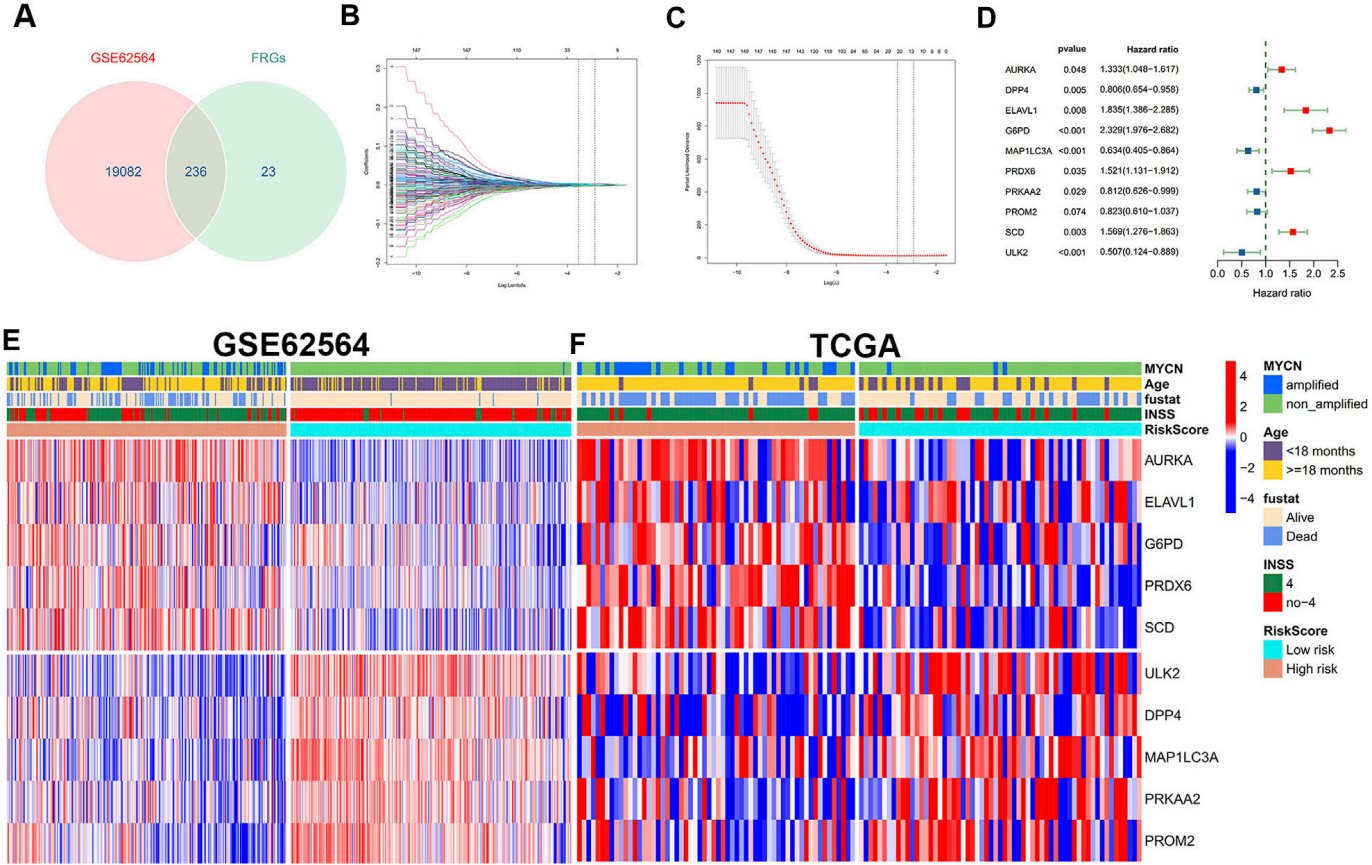
Construction and definition of the FRG signature. **(A)** Venn diagram to identify 236 FRGs in NB patients from the GSE62564 data set. **(B, C)** LASSO Cox regression model constructed from the 149 signature FRGs. Optimal lambda value is 0.02880196. According to the best fit contour, a 21-FRGs group was determined to the next step. **(D)** Multivariate Cox regression analysis confirmed independent prognostic factors which included 10 FRGs with HRs and *p* values. Grouping and heatmap of patients in GSE62564 **(E)**, TCGA **(F)**.

A brief flowchart is shown in Figure 1. In the GSE62564 data set, after performing the match between the Ensembl ID and mRNA annotation file, 236 FRG mRNAs were sorted out by intersecting the FRG list (Figure 2A). First, a univariate Cox regression analysis was performed to single out genes associated with patient survival. Then, by *p* < 0.01, there were 149 FRGs selected in NB patients. The LASSO Cox regression model and multivariate Cox regression were applied to find key genes that were most associated with the prognosis of NB (Figures 2B–D). Subsequently, a gene-based prognostic model of 10 FRGs (AURKA, DPP4, ELAVL1, G6PD, MAP1LC3A, PRDX6, PRKAA2, PROM2, SCD, and ULK2) was established to evaluate the risk of patients as described by the abovementioned methods. The risk score of the FRG signature named RiskScore was calculated from the expression of the 10 genes and the relative coefficient. Additionally, NB patients were divided into the low-risk and high-risk groups on the basis of their median RiskScore. Ordering by RiskScore in the training cohort (GSE62564) and the validation set (TCGA), heatmaps were shown to present the different expression levels of the 10 genes and clinical information (Figures 2E, F). In the training set, with an increase in RiskScore, the expression levels of AURKA, ELAVL1, G6PD, PRDX6, and SCD were upregulated. Meanwhile, ULK2, DPP4, MAP1LC3A, PRKAA2, and PROM2 were distinctly downregulated. MYCN-amplified status, INSS stage IV, and death were enriched in the high-risk group. The validation cohort showed similar levels of genes and clinical information. These results have indicated that the high RiskScore positively correlated with NB malignancy. Meanwhile, the characteristics of NB patients in the training and validation cohorts are shown in Supplementary Figure S1.

**FIGURE 3:**
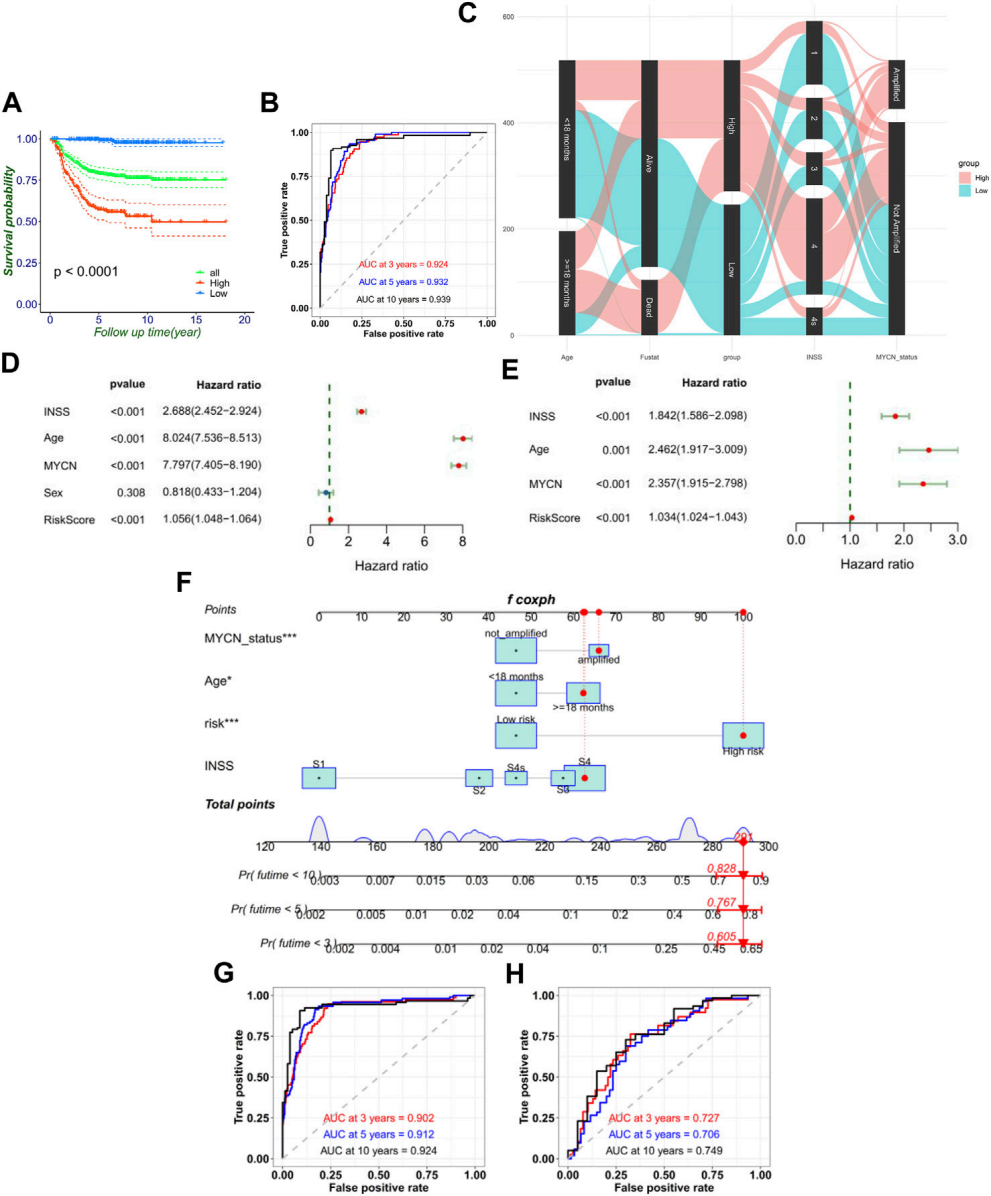
FRG signature’s ability of prognosis prediction and the construction of nomogram. **(A)** Signature could divide the overall prognosis into the high-risk and low-risk groups. **(B)** Time-dependent ROC curve of the RiskScore in the training cohort. **(C)** Sankey diagram directly shows the relationship of the signature with age, MYCN status, INSS stage, and mortality. Forest plot of univariate **(D)** and multivariate **(E)** Cox regression results of the RiskScore and clinical variables. **(F)** Establishment of the nomograms which include the RiskScore and clinical variables. ROC curves of the model for predicting the 3-, 5-, and 10-year survival in the training cohort **(G)** and external validation cohort (TCGA) **(H)**.

The Kaplan–Meier survival curves for the 10 hub FRGs are shown in Supplementary Figure S2. Besides, the Kaplan–Meier curves for the training set shows that the low-risk group had significantly longer OS than the high-risk group in NB (Figure 3A). The consistency of results was validated for the TCGA data set (Supplementary Figures S3A, B). Meanwhile, the 3-, 5-, and 10-year calibration plots for the probability of survival showed optimal agreements between observation and prediction (Supplementary Figure S3). The ROC curve was used to predict the 3-, 5-, and 10-year survival of NB patients. The signature of the 10 FRGs exhibited striking prognostic validation, with the AUC values of 0.924, 0.932, and 0.939 in GSE62564, and 0.709, 0.709, and 0.765 in TCGA databases (Figure 3B; Supplementary Figure S3C). Besides, we combined the RiskScore levels (high vs. low), age (<18 months vs. ≥18 months), INSS stages (I–IV, IVs), MYCN status (amplified vs. not amplified), and vital status to draw a comprehensive Sankey diagram, from which we explored that the low-risk group corresponded to a younger age, better MYCN status, earlier INSS staging, and very low mortality, while the high-risk group corresponded to completely the opposite (Figure 3C). These results have illustrated that the signature of the 10 FRGs is a reliable prognostic indicator in NB.

### FRG signature serves as valuable marker for clinical prognostic targets

Moreover, univariate Cox regression and multivariate Cox regression of the FRG signature RiskScore was performed in the training set (*p* < 0.001, univariate Cox regression; *p* < 0.001, and multivariate Cox regression). It verified the independence of the clinical prognostic significance of the RiskScore (Figures 3D, E). The consistency of results was also validated in the validation set. Then, a 3-, 5-, and 10-year survival nomogram prediction model was built with independent prognostic factors for the OS of patients in the training set (Figure 3F). The C-index was 0.865. We selected the patient GSM1529160. Then, the INSS stage S4 equaled 63 points, ≥18 months equaled 62 points, MYCN-amplified equaled 66 points, and high risk equaled 100 points by the nomogram. In total, the chosen sample equaled 291 points with the prediction values of 0.605, 0.767, and 0.828 for OS <3, <5, and <10 years. In fact, the chosen patient’s OS was 4.64 years. Using the ROC curve to predict the 3-, 5-, and 10-year survival of NB patients, the AUC values were, respectively, 0.902, 0.912, and 0.924 in GSE62564 and 0.727, 0.706, and 0.749 in TCGA database (Figures 3G,H). This meant that RiskScore combined with prognostic clinical features showed a good predictive value.

### Association with MYCN status

**FIGURE 4:**
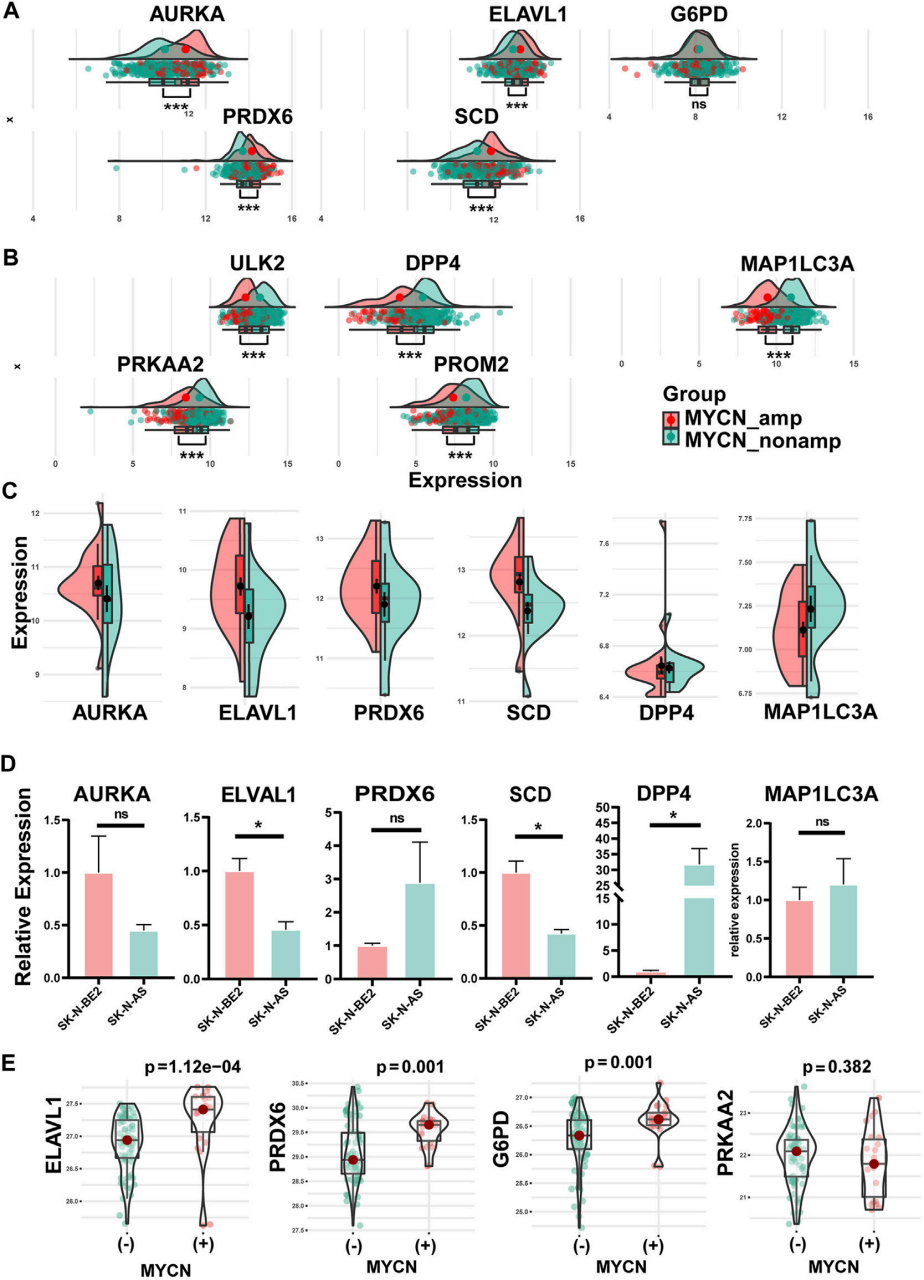
Differential expression of hub FRGs between MYCN amplification and MYCN non-amplification in tissue samples and cell lines and verification in qRT-PCR. **(A)** Differential expression of protective FRGs in tissue samples between MYCN amplification and MYCN non-amplification. **(B)** Differential expression of risk FRGs in tissue samples between MYCN amplification and MYCN non-amplification. Consistent with the expression trends in the tissue, differential expressions of AURKA, ELAVL1, PRDX6, SCD, DPP4, and MAP1LC3A between MYCN amplification and MYCN non-amplification in NB cell lines **(C)**. **(D)** Differential expressions of six FRGs among SK-N-AS (MYCN–non-amplified) and SK-N-BE2 (MYCN-amplified). **(E)** Differential protein expression of four FRGs among tissues with different MYCN statuses.

As a significant prognostic factor in NB, MYCN proto-oncogene amplification consistently predicts malignant diseases. So, the MYCN status was performed to explore the different gene expressions of each FRG of the signature. Except for the *p*-value of G6PD that was not significant, AURKA, ELAVL1, PRDX6, and SCD had higher expression levels in the MYCN-amplified set than in the MYCN–non-amplified set in the training group (345 patients: 58 MYCN-amplified, 287 MYCN–non-amplified) (*p* < 0.001) (Figure 4A). Meanwhile, ULK2, DPP4, MAP1LC3A, PRKAA2, and PROM2 showed the opposite trends (*p* < 0.001) (Figure 4B). Similar trends were revealed in the validation cohort (Supplementary Figures S4A, B). Furthermore, in Figure 4C, the NB cell line data set showed that AURKA, ELAVL1, PRDX6, and SCD were significantly enriched in the MYCN-amplified cell lines, whereas DPP4 and MAP1LC3A were enriched in the MYCN–non-amplified cell lines.

We performed qRT-PCR applying two cell lines: SK-N-AS (MYCN–non-amplified) and SK-N-BE2 (MYCN-amplified). The six genes (Figure 4C) screened with the cell line and tissue database were verified by experiments. Experimentally, these genes mostly showed the same trend as the NB cell lines database revealed, except for PRDX6. There were significant differences in the expressions of ELAVL1, SCD, and DPP4 and insignificant differences in the expressions of AURKA, MAP1LC3A, and PRDX6 between the SK-N-AS (MYCN–non-amplified) and SK-N-BE2 (MYCN-amplified) cell lines (Figure 4D).

Through the analysis of protein sequencing results, we obtained a total of 6,389 proteins, of which four proteins corresponded to the genes in the signature, namely, G6PD, PRDX6, ELAVL1, and PRKAA2. The results (Figure 4E) showed that G6PD, PRDX6, and ELAVL1 were highly expressed in MYCN(+) (*p* < 0.05); PRKAA2 was highly expressed in MYCN(−) (*p* > 0.05).

### Functional enrichment analyses

**FIGURE 5:**
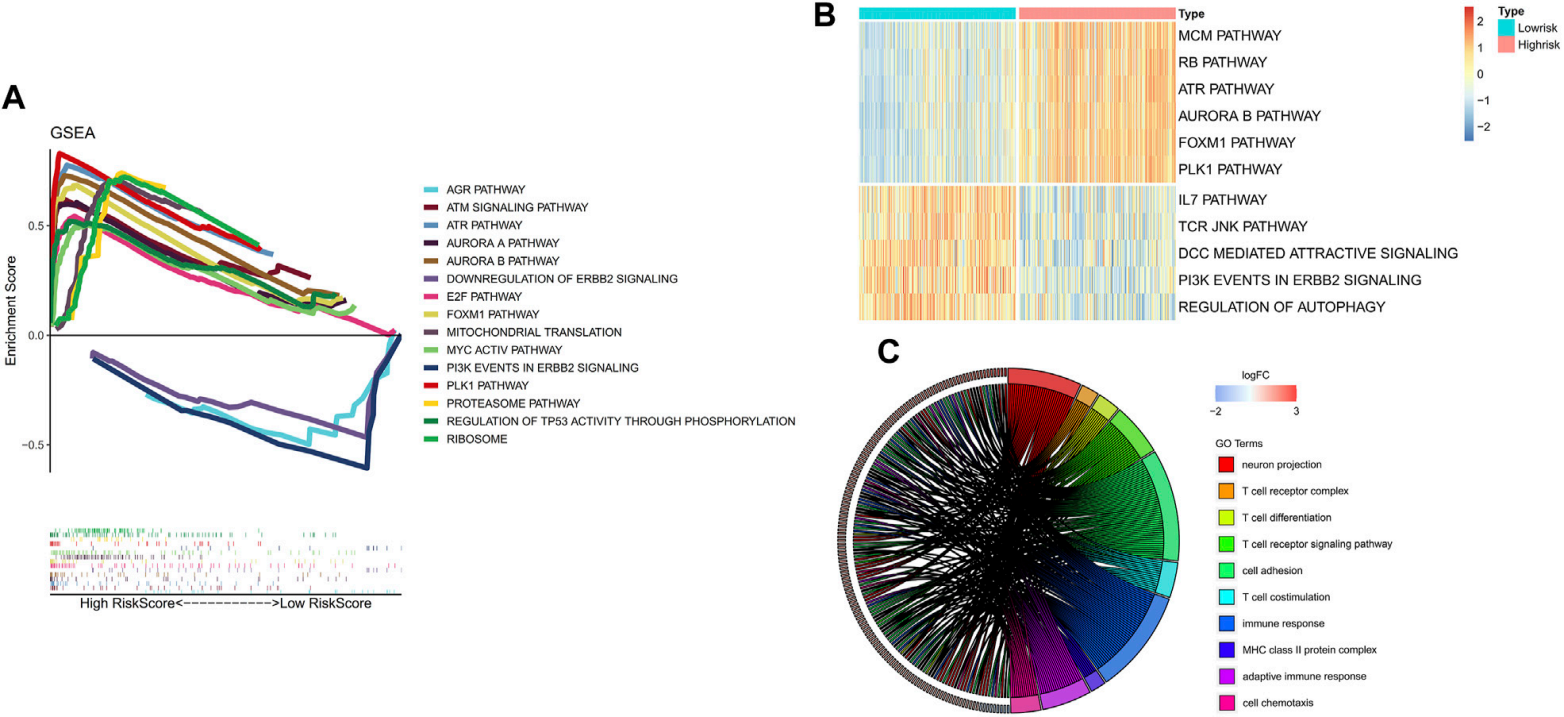
Results of functional and pathway analysis for the FRG signature. **(A)** Significantly enriched pathways by GSEA. **(B)** Significantly enriched pathways by GSVA. **(C)** Significant results of functional analysis of GO terms.

To clarify the potentially functional signature characteristics of the FRG signature in NB, we conducted GSEA to analyze the differences between the enriched gene sets. Setting *p* < 0.05 as the cutoff value, we found that multiple autophagy- and immunity-associated pathways were involved (Figure 5A), indicating that lower RiskScores were associated with antitumor immunity, which included the downregulation of the ERBB2 signal pathway. Yet, a higher RiskScore was associated with the aurora A and B pathways. Subsequently, the GSVA was applied for validation. Consistent with the GSEA results, it was shown that the RiskScore was markedly associated with autophagy- and immunity-associated pathways (Figure 5B). In the GO analysis, the DEGs were remarkably enriched in neuron projection, MHC class II protein complex, cell chemotaxis, and T-cell–related immune response (Figure 5C). Thus, we found that the FRG signature leads to a very differential characteristic of the TME immune cells infiltration phenotype.

### Immune landscape of FRG signature

**FIGURE 6:**
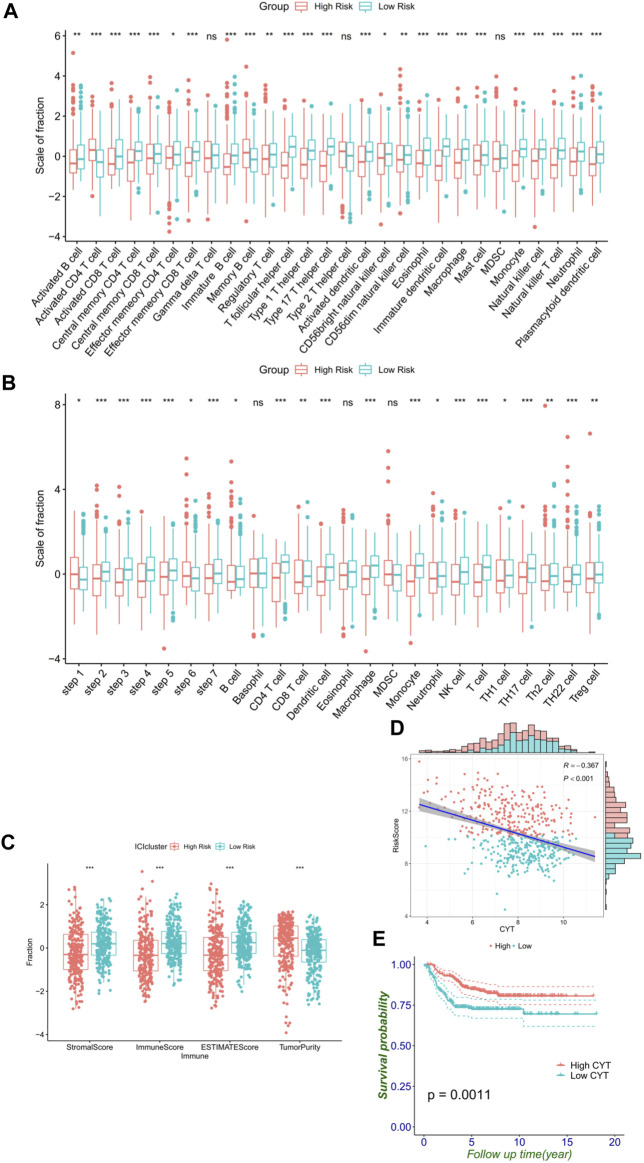
FRG signature reflects immune cell activation. **(A)** Histogram shows the normalized absolute abundance of 28 immune cell categories in individual samples of high- and low-risk groups. **(B)** TIPs to estimate the activity scores and major immune-related cells in tumor tissues. **(C)** Degree of the stromal, immune, estimate score, and tumor purity in high- and low-risk groups. **(D)** Relationship between the RiskScore and CYT. **(E)** Level of the CYT index significantly distinguishes survival.

Since prior work has demonstrated that functional enrichment is an immune-related function, we explored whether the RiskScore of the FRG signature was correlated with NB immunity. With the immune score defined by 28 categories of immune cells using the ssGSEA algorithm, the analysis of immune cell infiltration illustrated the abundance of innate immune cell infiltration such as natural killer cell, macrophage, mast cell, plasmacytoid dendritic cell, and eosinophil in the low-risk group. Meanwhile, specific immune cells were abundant, namely, CD8 T cell, immature B cell, T follicular helper cell, T helper cell except activated CD4 T cell, and memory B cell, in the low-risk group (Figure 6A). Accordingly, an important index named the seven-step cancer-immunity cycle was evaluated for the status of anti-cancer immunity. In the high-risk group, activities of various steps in the cycle were seen to be upregulated such as the release of cancer cell antigens (Step 1) and recognition of cancer cells by T cells (Step 6). Whereas in the low-risk group, cancer antigen presentation (Step 2), priming and activation (Step 3), trafficking of immune cells to tumors (Step 4, such as CD4 T cell, CD8 T cell, NK cell, dendritic cell, B cell, Treg cell, and TH1 cell), infiltration of immune cells into tumors (Step 5), and killing of cancer cells (Step 7) were stronger than that in the high-risk group. These elevated activities of the steps showed potent immunological potential (Figure 6B). In the training set, the high-risk group showed significantly lower stroma, immune, and ESTIMATE scores, but higher tumor purity than the low-risk group (Figure 6C).

As a result, high CYT was associated with the low-risk group and better survival (Figures 6D,E), which reflected that the low-risk group had a stronger immune-mediated attack against cancer cell function. These differential analyses between the two risk subgroups were shown to be the same in the validation cohort, and similar results were observed in the validation cohort (Supplementary Figure S5).

### FRG signature could predict immunotherapy benefit

**FIGURE 7:**
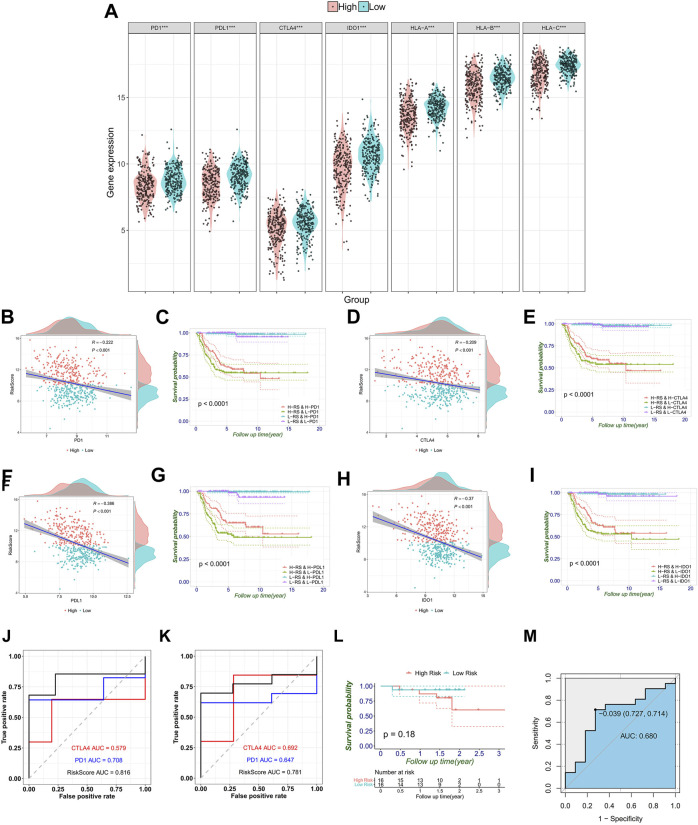
Correlation between immune checkpoint genes, HLA genes, and RiskScores, and validation of the signature in melanoma immune therapy data. **(A)** Immune checkpoint gene expression that includes PD-1, PD-L1, CTLA4 and IDO1, and HLA-ABC in the high- and low-risk groups stratified by the risk signature. **(B–I)** Respective correlation between the RiskScore and individual immune checkpoint gene, and the combination divides prognosis into four groups with significant differences (*p* < 0.0001). Comparison of ROC curves with PD-1 and CTLA4 shows the superiority of the FRG signature in predicting overall survival **(J)** and progression-free survival **(K)**. **(L)** Generally, melanoma patients are divided into high- and low-risk groups. **(M)** This AUC showed the predictive value of the RiskScore for the response of immunotherapy.

Subsequently, the immune checkpoints (PD-1, PD-L1, CTLA-4, and IDO-1) and human leukocyte antigen (HLA-A, HLA-B, and HLA-C) were upregulated in the low-risk group (Figure 7A). As shown in Figures 7B, D, F, H, the RiskScore of the FRG signature was significantly negatively correlated with immune checkpoint genes (r = −0.222 and *p* < 0.001 for PD-1, r = −0.386 and *p* < 0.001 for PD-L1, r = −0.209 and *p* < 0.001 for CTLA-4, and r = −0.37 and *p* < 0.001 for IDO-1). The expression of immune checkpoints affected the prognosis of patients (Supplementary Figure S6). In addition, to further investigate the effect of crosstalk between RiskScore and immune checkpoints on survival, patients were stratified into four parts based on the combination of RiskScore and immune checkpoints. Survival comparisons revealed that the RiskScore could distinguish the outcomes of NB with similar levels of immune checkpoint genes. Furthermore, patients with a low RiskScore and high expression level of immune checkpoints illustrated markedly longer survival rates than those with a high RiskScore and high expression level of immune checkpoints (*p* < 0.0001 for PD-1, PD-L1, CTLA-4, and IDO-1) (Figures 7C, E, G, I).

Furthermore, the abovementioned observed associations have led us to hypothesize that the RiskScore may be predictive of the response to immunotherapy. So, we tested the predictive value of the RiskScore in the melanoma combined anti-PD-1 and anti-CTLA-4 immunotherapy data sets. In OS, the RiskScore achieved an AUC of 0.816, which is comparable with the immune checkpoint gene markers (0.579 for CTLA-4 and 0.708 for PD-1) (Figure 7J). Moreover, the discriminative ability of the RiskScore was also observed to be higher in progression-free survival (PFS) than in CLTA-4 and PD-1. The AUC increased from 0.692 (CLTA-4) and 0.647 (PD-1) to 0.781 (RiskScore) (Figure 7K). Concurrently, the Kaplan–Meier curves show that the prognosis of the high-risk group is worse, although the *p*-value was not significant due to limited cases (Figure 7L). These results have indicated that lower RiskScore values are associated with better OS and PFS in tumor patients receiving immunotherapy. To explore the association between the response to immunotherapy and RiskScores, the RiskScore achieved an AUC of 0.68 in predicting the response to immunotherapy (Figure 7M).

### Tumor somatic mutation in distinct RiskScore patterns

**FIGURE 8:**
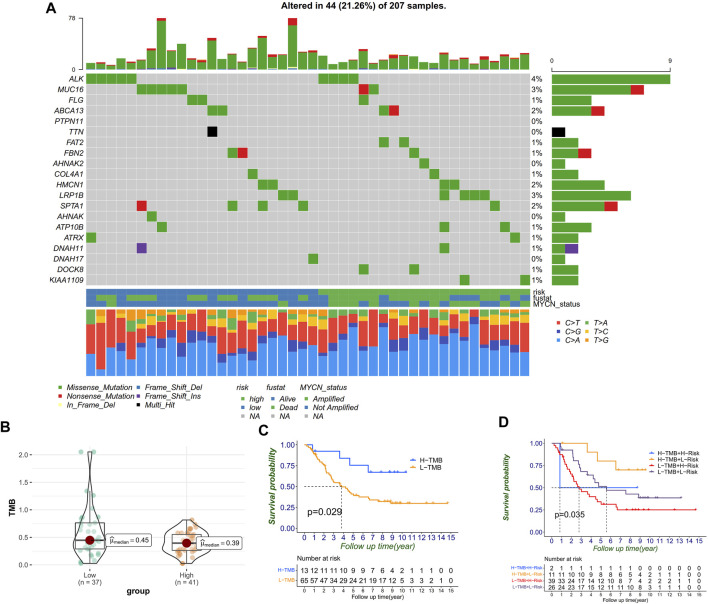
Correlation between RiskScores and TMB. **(A)** Mutation genes and types between high- and low-risk groups. **(B)** TMB level distribution between high- and low-risk groups. **(C)** TMB level significantly divided the prognosis. **(D)** TMB level, combined with RiskScore, can further divide prognosis into four groups.

We next classified the mutation data into various categories, where missense mutation occupied the most part, single-nucleotide variant (SNV) mutates most frequently, and C>A played the top type of SNVs in NB. Furthermore, we compared the mutational difference between the high- and low-risk groups and found that the high-risk group ALK mutated more than MUC16 in the low-risk group (Figure 8A). Moreover, we observed that TMB in the low-risk group was higher than it was in the high-risk group (Figure 8B). The Kaplan–Meier curves also proved the same trend, that the high-TMB group had significantly more OS than the low-TMB group in NB (Figure 8C). Moreover, patients with a low RiskScore and high TMB indicated significantly longer survival rates than those with high RiskScore and low TMB (*p* = 0.035) (Figure 8D).

### Identification of nodal genes and immunohistochemistry

**FIGURE 9:**
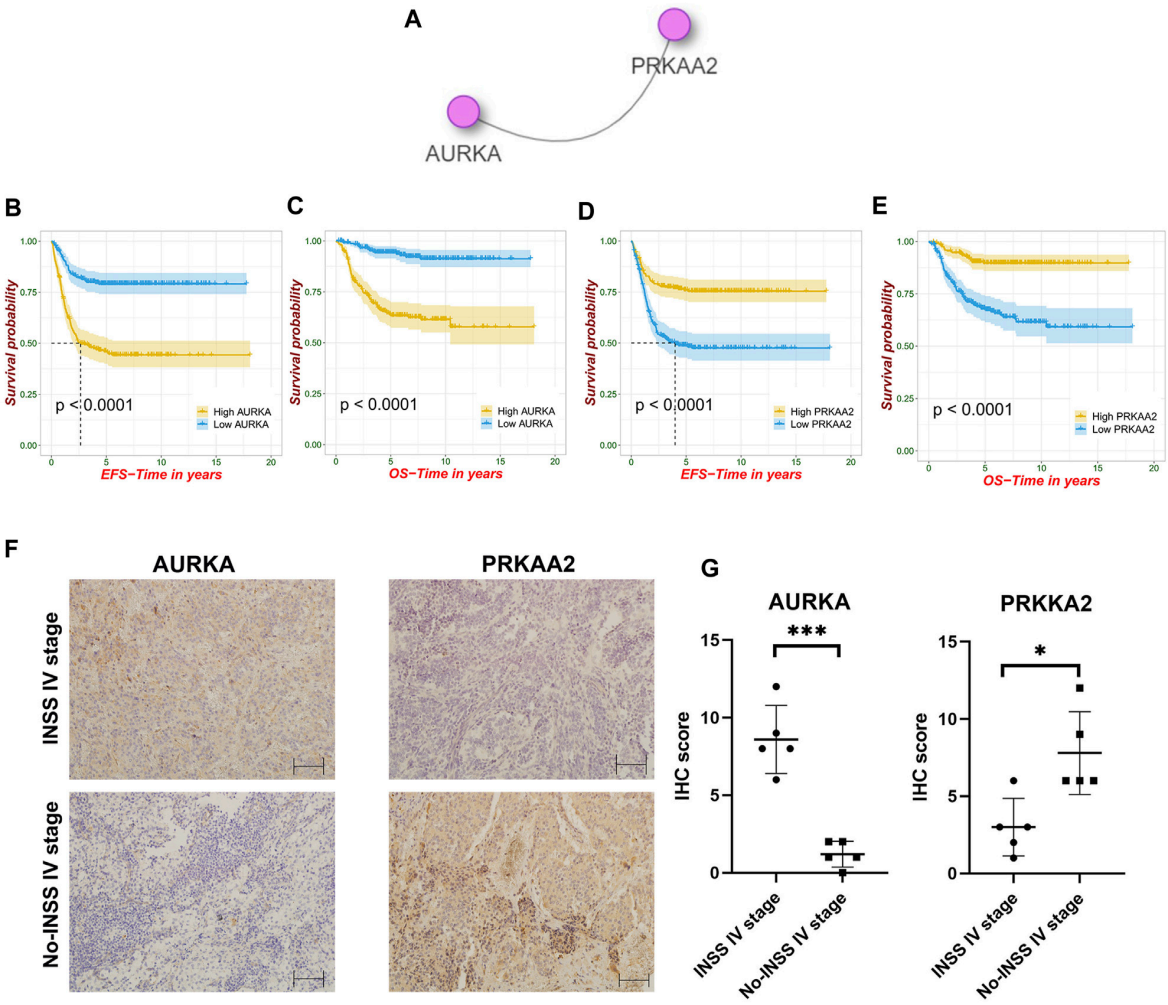
Identification of nodal FRGs and immunohistochemistry analysis. **(A)** Identification of two nodal genes specific in NB. **(B–E)** Survival analysis of AURKA and PRKAA2. **(F–G)** Different expression trends of AURKA and PRKAA2 in the INSS stage IV and non–INSS stage IV at ×200 magnification.

According to the integrated interactions database, we identified two key genes specific in NB, AURKA, and PRKAA2 (Figure 9A). In the survival analysis, high AURKA expression showed worse OS and EFS. Nevertheless, PRKAA2 was the opposite of AURKA (Figures 9B–E). The results confirmed the previous analysis in that AURKA was a risk factor and PRKAA2 was a protective one. Besides, at ×200 magnification, AURKA was expressed significantly higher in the INSS stage IV than in the other stages in the IHC analysis, whereas PRKAA2 was the opposite of AURKA (Figures 9F, G).

### Correlation between FRGs and T cells in NB microenvironment

**FIGURE 10:**
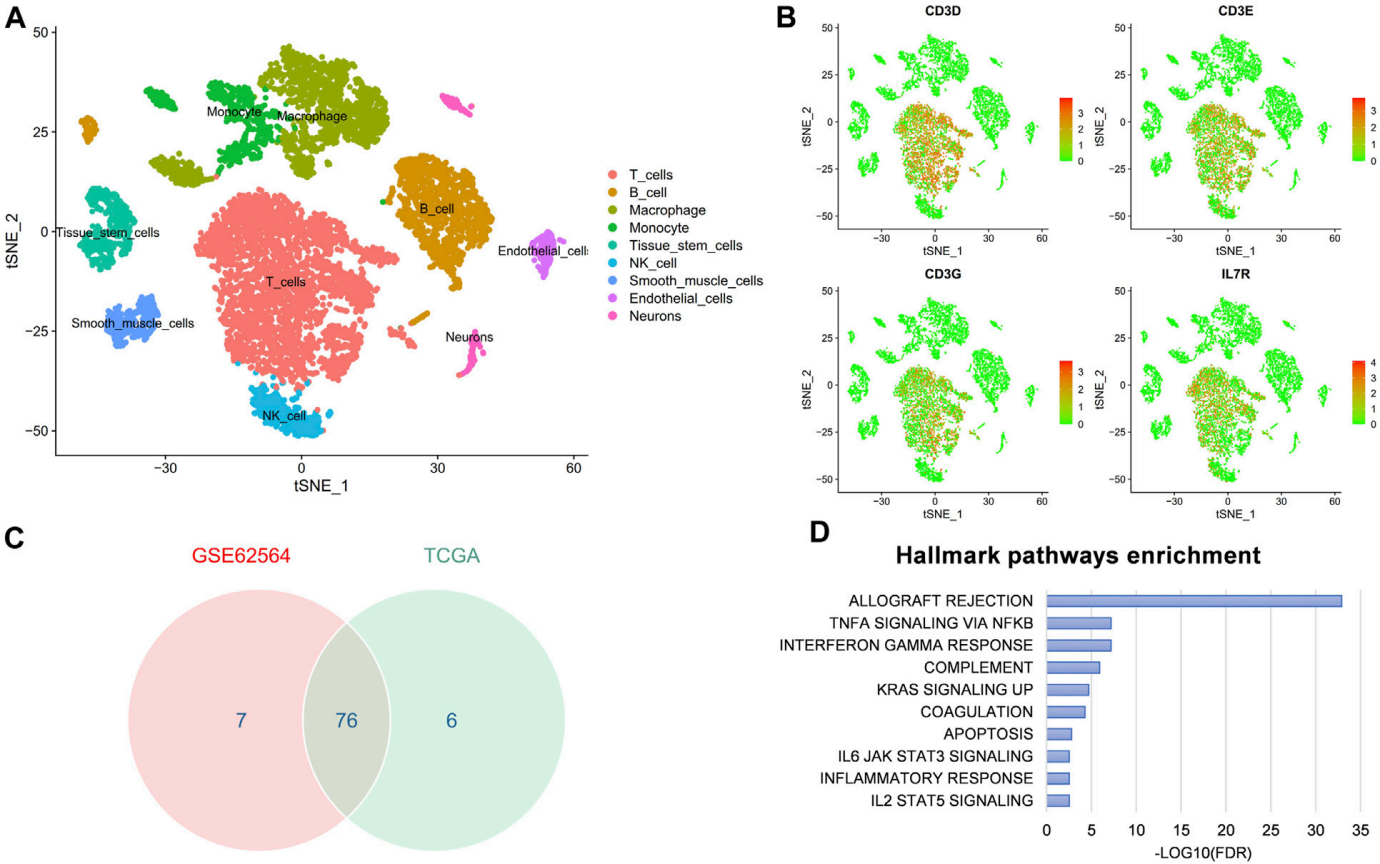
scRNA-seq reveals the correlation between FRG signature and T-cell marker genes in NB microenvironment. **(A)** t-SNE plot of cells from the NB microenvironment. **(B)** Distribution characteristics of major marker genes for T cell. **(C)** Venn diagram to identify 76 significantly associated genes. **(D)** GSEA hallmark analysis obtained from genes in the intersection.

As previously mentioned, our FRG signature could predict immunotherapy benefits and well distinguish the TME, so we further explored the correlation with T-cell killing at the single-cell level. First, the t-SNE plot showed the immune components in the NB microenvironment (Figure 10A) and second, the expression of the major marker genes (CD3D, CD3E, CD3G, and IL7R) for T cell (Figure 10B). We next took 76 intersection genes of the Spearman’s coefficient correlation >0.2 between the RiskScore and T-cell marker genes in the GSE62564 and TCGA data sets (Figure 10C). The pathway analysis revealed that the TNFA signaling via NFKB and interferon-γ response were regulated (Figure 10D).

## Discussion

As one of the most lethal childhood malignancies, NB presents clinicians with challenges and difficulties (Hara, 2012). There are multifarious means of immune escape, such as infiltrating immunosuppressive cells, modulation of antigen presentation machinery (APM), and secreting immunosuppressive factors (Vanichapol et al., 2018). The process to awaken the immune action in the microenvironment of NB has been considered a novel means to activate the effect of immunotherapy (Carlson et al., 2013). Ferroptosis is associated with the MYCN gene and might be closely related to shaping the tumor immune microenvironment (Angeli et al., 2019; Lu Y. et al., 2021). Our results also implied that the hub FRGs with an independent prognostic value might act as downstream bioactive molecules of MYCN onco-promotor gene in terms of the expression trends (Figure 4). Besides, immunotherapy for NB requires novel targets and powerful estimating models, so we developed this hub FRG–based prognostic signature and revealed the association between the signature and diverse immunophenotypes in NB.

After screening with LASSO and Cox regression, FRGs which included five protective genes, namely, ULK2, MAP1LC3A, DPP4, PROM2, and PRKAA2 and five risk genes, namely, AURKA, SCD, PRDX6, G6PD, and ELAVL1 were identified as the component genes of the FRG signature. In addition to tumor tissues in training and validation cohorts, we divided the cell line data according to the MYCN-amplification status, and the differential expressions of the abovementioned FRGs were compared. Subsequently, based on cell line data analysis, our PCR results revealed the potential relationship between six key genes and the MYCN gene. MYCN oncogene, as a promoter, may significantly drive the expression of a variety of oncogenes, such as AURKA and SCD (Huang and Weiss, 2013; María et al., 2017). Meanwhile, the expression trend of PRDX6 was inconsistent with the overall cell line data, but after consulting the GSE19274 database (Cole et al., 2011), the PRDX6 of SK-N-AS cell line was highly expressed than that of SK-N-BE2. More cell lines should be included in the research and verification processes.

The ferroptosis level cannot represent the malignant potential of tumor development. In fact, targeting ferroptosis in cancer might be a double-edged sword (Chen et al., 2021a; Chen et al., 2021b). As Supplementary Figure S2 shows, genes that had the same promoting/suppressing effect on ferroptosis can have different effects on prognosis. For example, as key activators of autophagy and regulators of ferroptosis, ULK2 and MAP1LC3A (also known as LC3) both take part in diverse carcinogenesis. Promoting autophagy and ferroptosis may cooperatively induce drug sensitivity and inhibit the development of NB (Liu et al., 2019). Second, the prognostic role of DPP4 is controversial. However, it is not only in our study that higher DPP4 might be associated with better survival in various cancers such as head and neck malignancies (Javidroozi et al., 2012). Besides, DPP4 is related to activation of AMPK in neural cell line SK-N-MC, providing a new version for NB treatment (Kornelius et al., 2015). Third, when compared to that in normal tissues, PROM2 was significantly overexpressed in breast, lung, bone marrow, and ovarian cancers, whereas it was underexpressed in colon, esophageal, gastric, kidney, prostate, and skin cancers (Subbroto et al., 2020). The protective relationship between PROM2 and prognosis of NB was first revealed in our study and worthy of further experimental verification. Finally, PRKAA2 has been shown to inhibit cell proliferation through the p53/p21 pathway and through modulation of the expression of p27 (Jones et al., 2005). Besides, PRKAA2 knockout in liver cancer enhanced tumor inflammation, also associated with the immune microenvironment (Qiu et al., 2019). The overexpression of PRKAA2 in NB could promote ROS production and cell apoptosis after bupivacaine treatment (Lu et al., 2011). Our screening of hub genes has also revealed the key node role of PRKAA2, which had significant impact on both event-free survival and overall survival. Besides, the results showed the molecular localization of PRKAA2 and might be further helpful for the development of target therapy in NB.

On the contrary, AURKA shows significantly higher expression in cancer tissues than in normal control tissues for multiple malignancies according to the TCGA database (Du et al., 2021). The silencing of AURKA is effective in antitumor efficacy of NB *in vitro* and *in vivo* (Yang et al., 2020). From the perspective of immunity, natural killer–derived exosomal miR-186 can directly inhibit the expression of AURKA and simultaneously inhibit growth, spreading, and TGFβ-dependent immune escape mechanisms in NB (Neviani et al., 2019; Schmittgen, 2019). Moreover, nodal gene results have shown that other than PRKAA2, AURKA was one of the nodal FRGs that had an important relationship with NB. Understanding the physical and chemical properties and its distribution in the cytoplasm and nucleus would be helpful to explore therapeutic targets for advanced disease (Ruijuan et al., 2021). Second, SCD may promote carcinogenesis while the inhibition of SCD can help rescue rapid alpha-synuclein toxicity in a neural cell model and affect αS homeostasis and toxicity in neuronal cells (Imberdis et al., 2019; Terry-Kantor et al., 2020). However, the role of SCD in NB prognosis was explored for the first time in the present study. Third, PRDX6 promotes the development of several cancer cells (Yun et al., 2014; Hu et al., 2020). Exogenous PRDX6 can rescue cellular damage induced by cellular hypoxia (CoCl_2_) chemically and significantly decrease CoCl_2_-induced apoptosis in SK-N-SH human NB cells (Asuni et al., 2015). Fourth, G6PD takes part in neuronal differentiation in the SH-S5Y5 cell line, meanwhile the aberrant activation of G6PD leads to enhanced cell proliferation and adaptation in many types of cancers (Almeida et al., 2018; Yang et al., 2019). Hence, we speculated that G6PD may be associated with the adverse biological behavior of NB cells. Finally, ELAVL1 might act as a part of the central oncogenic driver for malignant peripheral nerve sheath tumors (Palomo-Irigoyen et al., 2020). Moreover, activating ELAV may play a role in neurodegenerative diseases (Marchesi et al., 2016). Therefore, further experimental exploration of the mechanisms responsible for the poor prognostic effect of ELAVL1 in NB development is required.

The RiskScores calculated from FRG signatures were strongly correlated with clinical prognostic factors. In comparison, the Sankey diagram analysis revealed that a higher RiskScore was consistent with older age, MYCN amplification, and advanced INSS stage. Besides, as expected, the increase of the RiskScore might mean a higher relapse rate and worse prognosis. To further evaluate the prognosis, we used Cox regression to screen and construct a nomogram that included the RiskScore and clinically independent prognostic factors, which can accurately calculate long-term prognosis (until 10-year survival) of children with NB. External data validation also revealed that it had a satisfied prediction ability.

The GO analysis significantly revealed the relationship of the present signature with T-cell development and adaptive immunity, as well as neuron projection and cell adhesion that might participate in the development of NB. After comparison with groups, some clues about carcinogenesis were revealed. For example, first, aurora kinase A and B were both reported to correlate with poor survival and MYCN expression in NB, and aurora kinases A may have a direct physical interaction with the MYCN protein (Otto et al., 2009; Hsieh et al., 2019). Moreover, researchers used gene enrichment analysis and found that the carbohydrate metabolic process, fatty acid metabolic process, lipid biosynthetic process, and response to hypoxia were associated with aurora kinase inhibition in Th-MYCN transgenic NB mice, which would simultaneously have potential effects on the regulation of autophagy and ferroptosis (Lu J. et al., 2021; Chen et al., 2021c; McLeod et al., 2021; Ni et al., 2021; Wang et al., 2021). Second, the FOXM1 pathway was discovered to be involved in the tumorigenicity of aggressive NB cells through the maintenance of the undifferentiated state (Wang et al., 2011). Besides, the overexpression of FOXM1 might lead to malignant phenotypes by directly upregulating genes such as AURKB and MYC or indirectly upregulating genes such as ZEB1 and ZEB2 (Katoh et al., 2013). Meanwhile, the inhibition of FOXM1 induced apoptosis by inhibiting the activation of PI3K and AKT in NB cell lines (Liao et al., 2020). Next, a positive feedforward regulatory loop between the PLK and MYC pathways was revealed (Xiao et al., 2016). Iliaki et al. (2021) have reported that PLK1 was involved in immune and neurological disorders such as Alzheimer’s disease. Furthermore, Grinshtein et al. (2011) screened and then suggested that PLK1 inhibitors could be an attractive candidate therapy for metastatic NB. On the other hand, immune-related pathways like the IL17 and TCR JNK pathways were found to be enriched in the low-risk group. The role of IL17 in malignancy disease remains controversial (Lin et al., 2015; Tsai et al., 2021). The research value of IL17 secreted from different cells in the NB microenvironment has to be confirmed by further experiments due to the limited research at present (Zhang et al., 2020). Besides, JNK, known as c-Jun N-terminal kinase, is activated when T-lymphocytes are stimulated with the T-cell receptor (TCR) and CD28 (Kirk et al., 1999). Furthermore, the activation of JNK may induce the death of tumor cells accompanied with the release of mitochondrial cytochrome C and increase in autophagy inducing factors (Yu et al., 2019; Zheng et al., 2020). In addition, the downregulation of the ERBB2 pathway was found to be significantly related with good prognosis and immunotherapy response in another nervous system tumor, glioma (Mei et al., 2021). Last but not the least, the regulation of the autophagy pathway was also found enriched in the low-risk group, which is consistent with previous reports and worthy of further research (Meng et al., 2020).

Since the functional enrichment results have revealed a close relationship with metabolism, autophagy, and immunity, ssGSEA was performed, which showed that there were more active immune components in the microenvironment of NBs in the low-risk group, such as activated CD8 T cell, natural killer cell, and neutrophil cell, providing us with the possibility of low-risk patients being included in the “hot tumor” group. In addition, estimates of tumor immune infiltration found that the low-risk group had higher stromal scores, immunization scores, and estimation scores, implying more active immunity. Meanwhile, the low-risk group had lower immune purity, which meant low malignancy and low invasiveness. Moreover, the CYT score, defined by granzyme A and perforin expression and reflecting the immune cell killing function, was successfully used for underlying immunity (Takahashi et al., 2020). We also hypothesized that CYT-high NBs had low risk in the present FRG signature and significantly better prognosis . Furthermore, we found that the low-risk group was more activated as a whole in tracking the analysis of the tumor immunophenotype, except step 1 release of cancer cell antigens and step 6 recognition of cancer cells by T cells (Liwen et al., 2018). Furthermore, the low-risk group had a higher expression of immune checkpoints and markers, such as PD-L1 and HLA-ABC, which is similar to several reports by Ming et al. (2021). Therefore, the results have supposed that patients in the low-risk group would benefit more from immune checkpoint blocking therapy. Moreover, we can accurately predict the treatment effect by further subdividing the KM curves.

Unfortunately, although NB is one of the most common childhood tumors, there is still no available data on the outcome of immunotherapy. However, melanoma with publicly available immunotherapy data shares several similar characteristics with NB. First, both of them share a common origin, arising from the neuroectodermal tissue, the portion of the ectoderm that gives rise to the central and peripheral nervous systems (Morandi et al., 2018). Second, NB and melanoma share common immune markers, such as GD2, a therapeutic target that has been carried out in both malignancies (Cheresh et al., 1986; Rakhmilevich et al., 2017; Tran et al., 2017). Moreover, Avitabile et al. (2020) have found that NB and melanoma share 1p13.2 as the susceptibility locus and SLC16A1 as the common oncogene by cross-disease meta-analysis of GWAS. Finally, in clinics, neuron-specific enolase (NSE) can aid the diagnosis of both melanoma and NB (Dhillon et al., 1982). The serum levels of cytoplasmic melanoma-associated antigen at diagnosis may predict clinical relapse in NB patients (Morandi et al., 2011). In addition, immune mechanisms underlying spontaneous regression in NB can predict melanoma response to immune checkpoint blockade (Auslander et al., 2018). Therefore, as Sha et al. (2022) have previously reported, we used melanoma data for immunotherapy validation. In the present study, the signature could classify the melanoma population into the high- and low-risk groups. The AUCs showed more predictive power of the signature for the prognosis. Moreover, the signature was capable of predicting immune checkpoint blocking responses.

Numerous reports have shown that tumor cells with higher TMB were more easily recognized by the immune system, and immunotherapy was more likely to respond (Maleki, 2018). A higher tumor mutation burden can also induce more antigens and anti-tumor immunity, which finally results in better prognosis in NB. Moreover, MUC16 and ALK mutation were common in the NB cohort, meanwhile MUC16 mutation was especially more common in the low-risk group. Li et al. (2018) have reported that MUC16 mutations might be associated with higher tumor mutation load, as well as better survival outcomes and immune response. Furthermore, the role of MUC16 mutation in NB is worthy of further explanation (Lee et al., 2020). Besides, as was reported, high ALK mutation in NB was associated with poor prognosis (Borenas et al., 2021). All of these findings have shown that the FRG signature not only distinguished the TME immune cell infiltration but also correlated with the mutation landscape, underling the significance of ferroptosis in NB development again. Moreover, scRNA-seq analysis showed that the signature was related to T cells, and TNFA signaling and interferon-γ might be the target pathway to overcome immunotherapy tolerance (Lorenzi et al., 2012).

There is no doubt that our research still has some limitations. Generally, a large sample of the clinical multicenter prospective cohort is required to verify our results and the clinical value of these genes still require further verification through follow-up research. Besides, the gene set used in this study cannot accurately represent the type and function of all immune cells, and the mRNA and protein levels cannot be accurately equivalent. Finally, the FRG signature showed a moderate predictive effect on immune checkpoint treatment of melanoma, which should further be verified in the data set of NB immune treatment in the future.

## Conclusion

In summary, our study established and validated an FRG-based signature, which could divide patients into high-risk and low-risk groups in multiple cohorts. Meanwhile, the RiskScore calculated by the signature showed a significant relationship with a variety of cell components of the immune microenvironment and immune checkpoint expression and could effectively predict the response to immunotherapy. Moreover, this study proposed many effective pathways and targets related to the biological behavior of NB.

## Data Availability

Publicly available data sets were analyzed in this study. These data can be found at http://ncbi.nlm.nih.gov/geo/; https://portal.gdc.cancer.gov/; https://hgserver1.amc.nl/cgi-bin/r2/main.cgi?option=login; http://www.zhounan.org/ferrdb/; http://tide.dfci.harvard.edu/; http://biocc.hrbmu.edu.cn/TIP/index.jsp; http://timer.cistrome.org/.

## Supplementary material

SUPPLEMENTARY FIGURE S1 Overall clinical data of the training cohort (GSE62564) and external validation cohort (TCGA).

SUPPLEMENTARY FIGURE S2 Kaplan–Meier survival curves of 10 hub FRGs. **(A)** In the ferroptosis driver genes; MAP1LC3A, ULK2, DPP4, and PRKAA2 were significantly positively correlated with favorable prognosis (p < 0.05), while ELAVL1 and G6PD were significantly negatively correlated with favorable prognosis (p < 0.05); **(B)** in the ferroptosis suppressor genes, PRDX6 and SCD were significantly positively correlated with favorable prognosis (p < 0.05), while PROM2 was significantly negatively correlated with favorable prognosis (p < 0.05); **(C)** ferroptosis marker gene AURKA was negatively correlated with a favorable prognosis.

SUPPLEMENTARY FIGURE S3 The FRG signature’s ability of prognosis prediction in TCGA. The signature could divide the overall prognosis into high-risk and low-risk groups **(A)**. Calibration curves of the signature for 3, 5, and 10 years in TCGA **(B)**. Time-dependent ROC curve of the RiskScore in TCGA **(C)**.

SUPPLEMENTARY FIGURE S4 Differential expression of hub FRGs between MYCN amplification and MYCN non-amplification in tissue samples of TCGA. **(A)** Different expressions of risk genes in tissue samples between MYCN amplification and MYCN non-amplification in TCGA cohort. **(B)** Different expressions of protective genes in tissue samples between MYCN amplification and MYCN non-amplification in TCGA cohort.

SUPPLEMENTARY FIGURE S5 FRG signature reflecting immune cell activation in TCGA. **(A)** Histogram shows normalized absolute abundance of 28 immune cell categories in individual samples of high- and low-risk groups. **(B)** TIP to estimate the activity scores and major immune-related cells in tumor tissue. **(C)** Degree of stromal, immune, estimate score, and tumor purity in high- and low-risk groups. **(D)** Relationship between the RiskScore and CYT. **(E)** Survival status correlated with the CYT index.

SUPPLEMENTARY FIGURE S6 Univariate Cox regression and Kaplan–Meier survival analysis on each immune checkpoint. **(A)** Univariate Cox regression of immune checkpoints. **(B–E)** Kaplan–Meier survival analysis on each immune checkpoint.

SUPPLEMENTARY TABLE S1 Primers information.
