# Supplementary figures and images for: Integrative analysis of multi-omics data for discovery of ferroptosis-related gene signature predicting immune activity in neuroblastoma
Source: Front Pharmacol. 2023 Jul 13;14:1162563. doi: 10.3389/fphar.2023.1162563 (PMC10373597; doi:10.3389/fphar.2023.1162563)

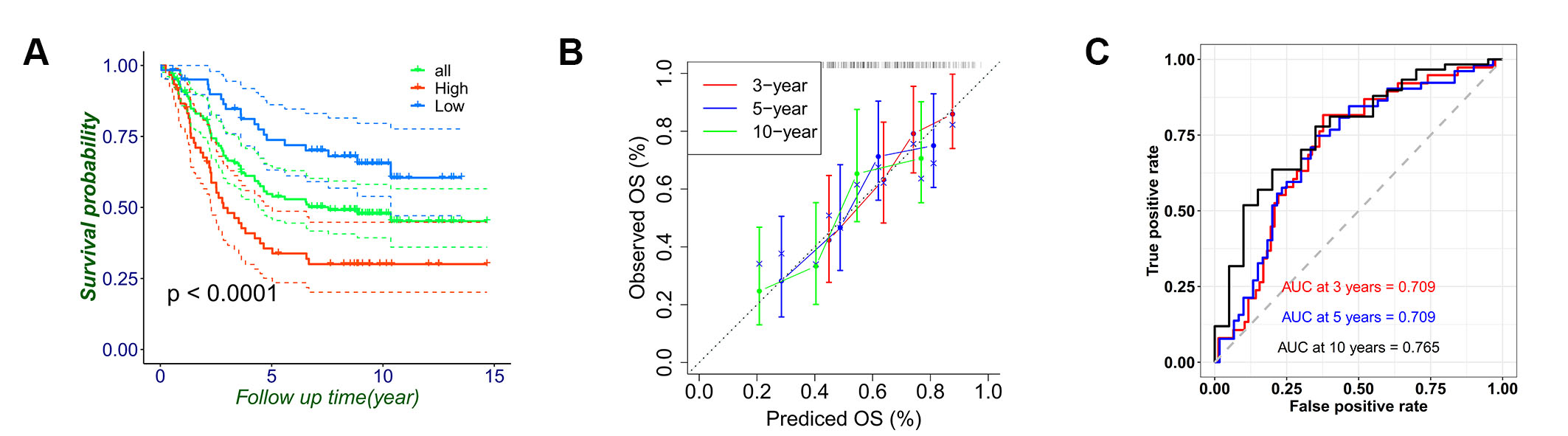

Supplement: Supplementary file 1 [file Image3.JPEG]

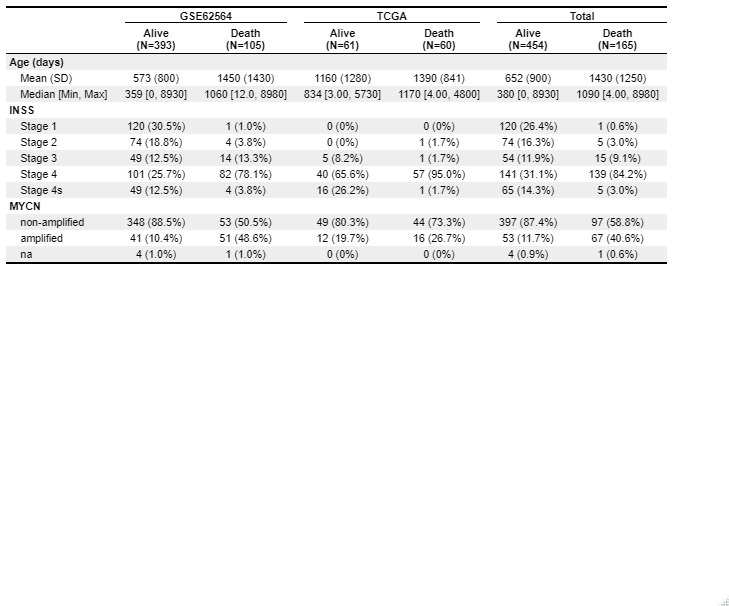

Supplement: Supplementary file 2 [file Image1.JPEG]

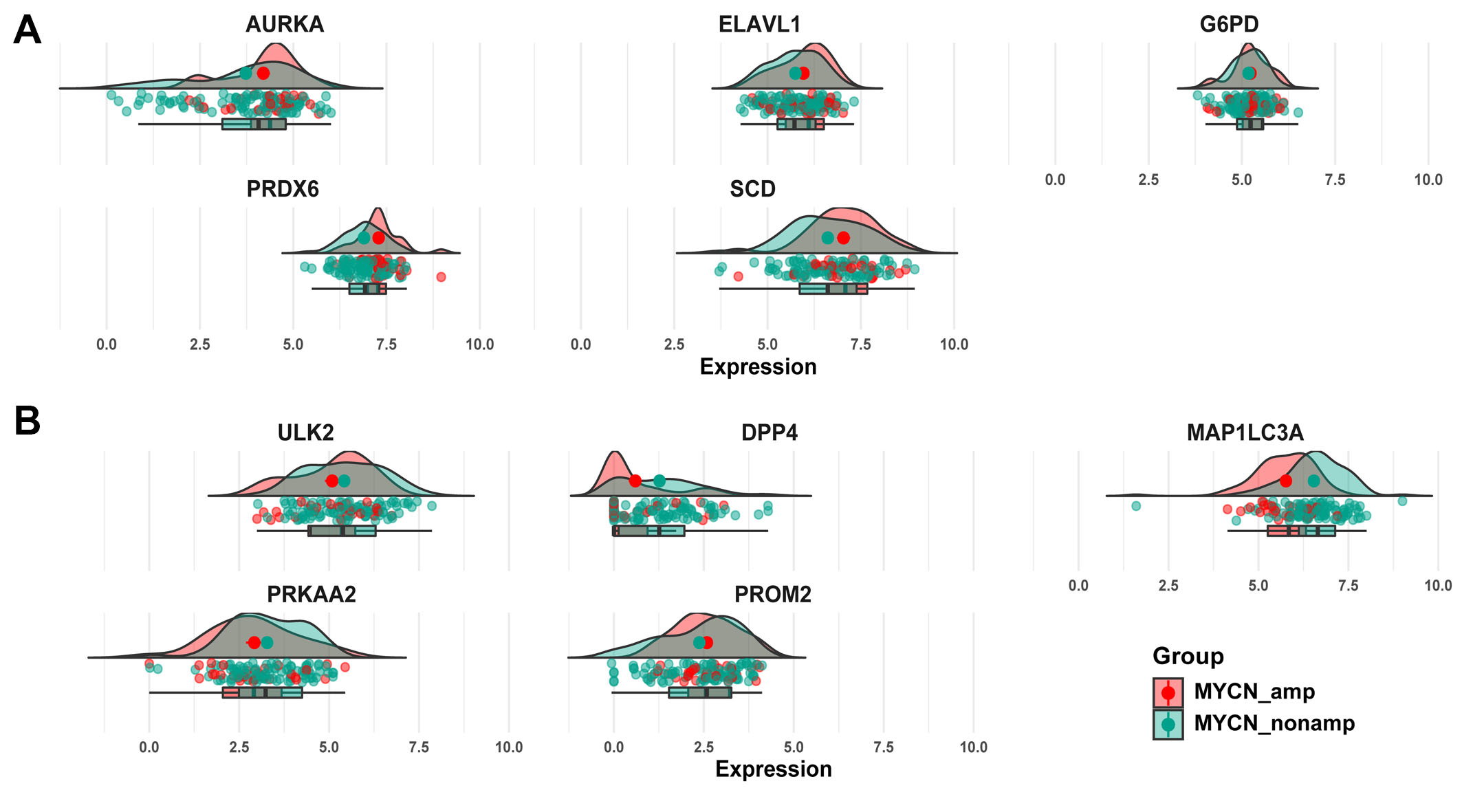

Supplement: Supplementary file 3 [file Image4.JPEG]

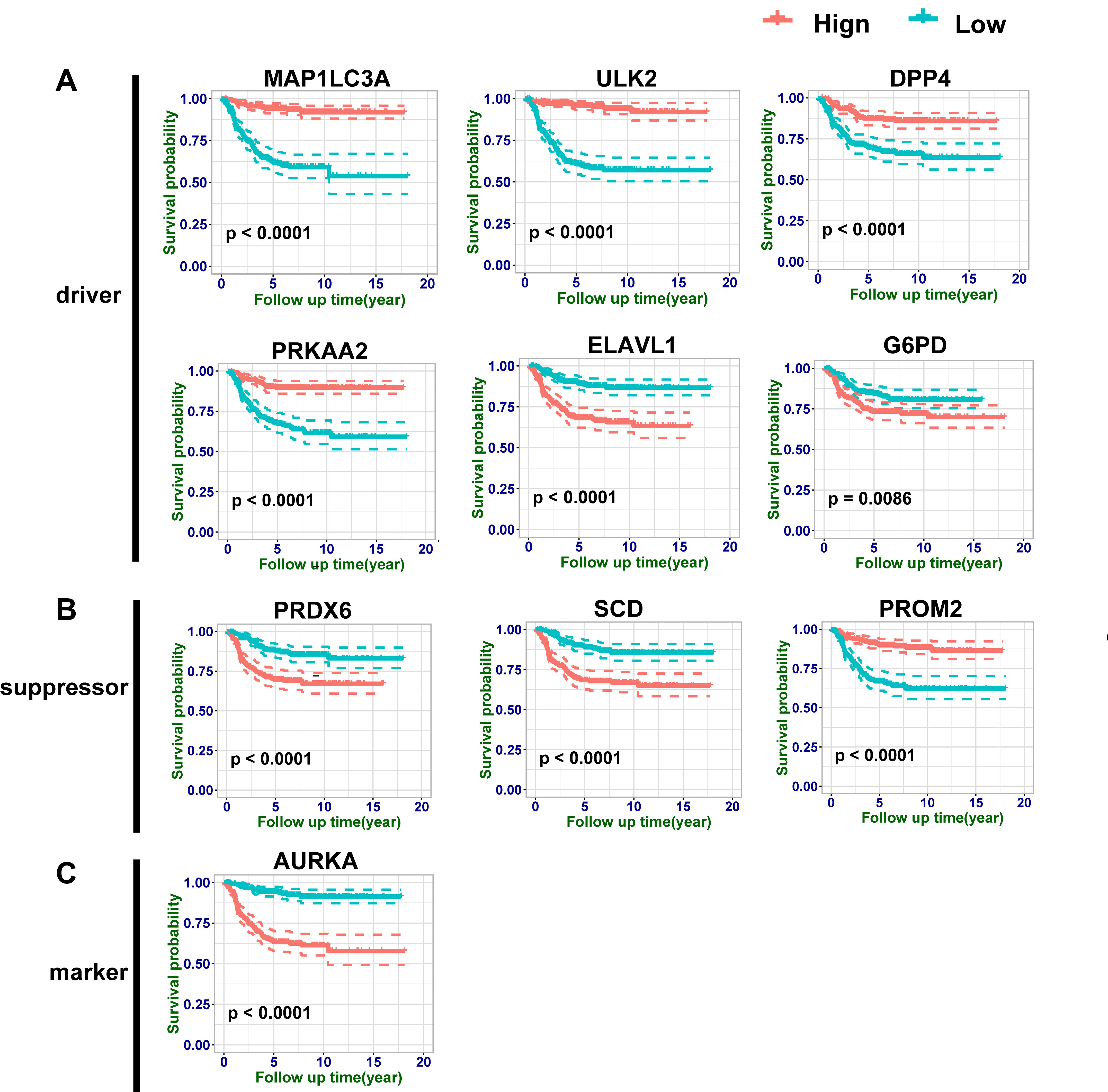

Supplement: Supplementary file 4 [file Image2.JPEG]

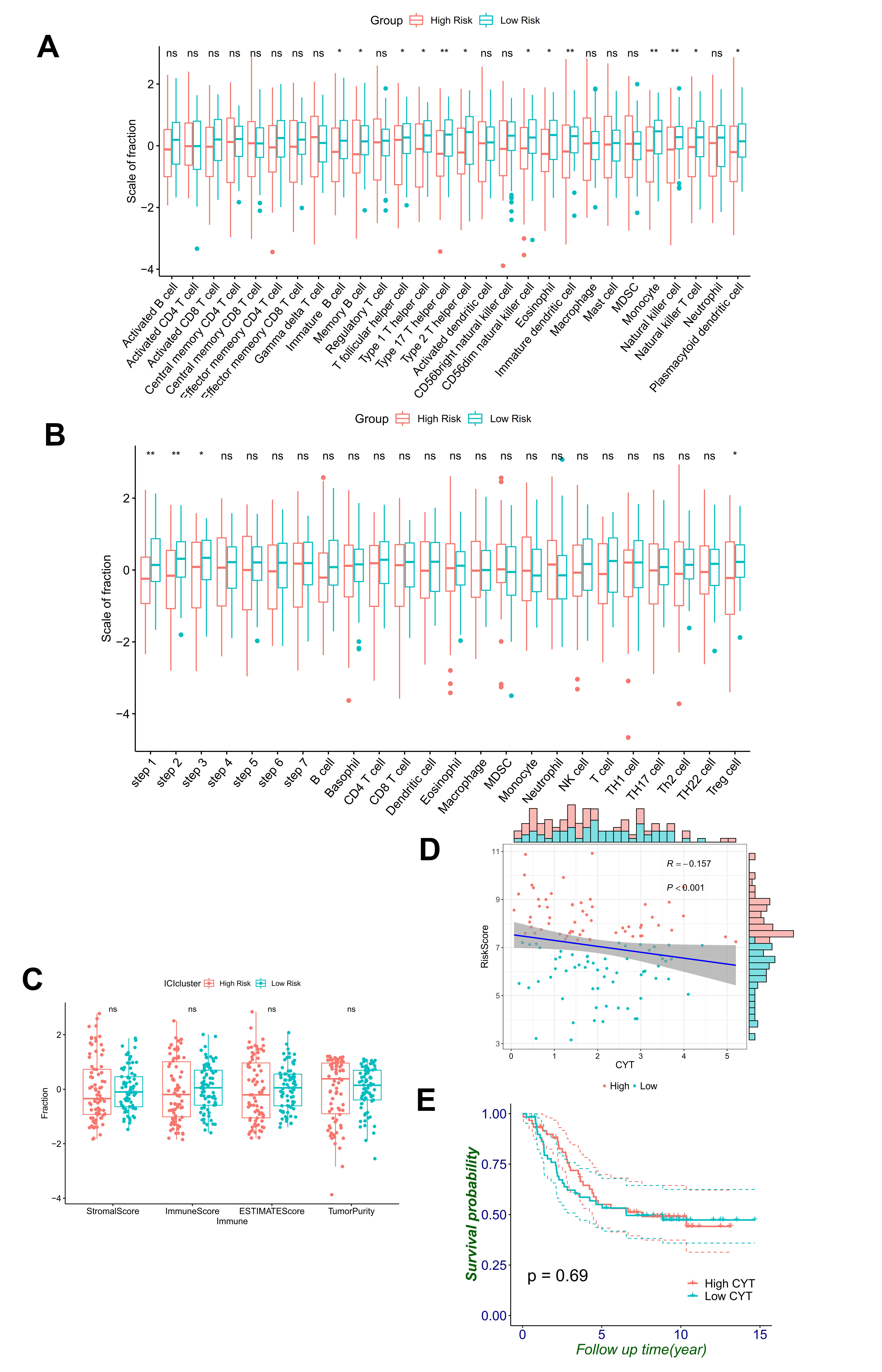

Supplement: Supplementary file 5 [file Image5.JPEG]

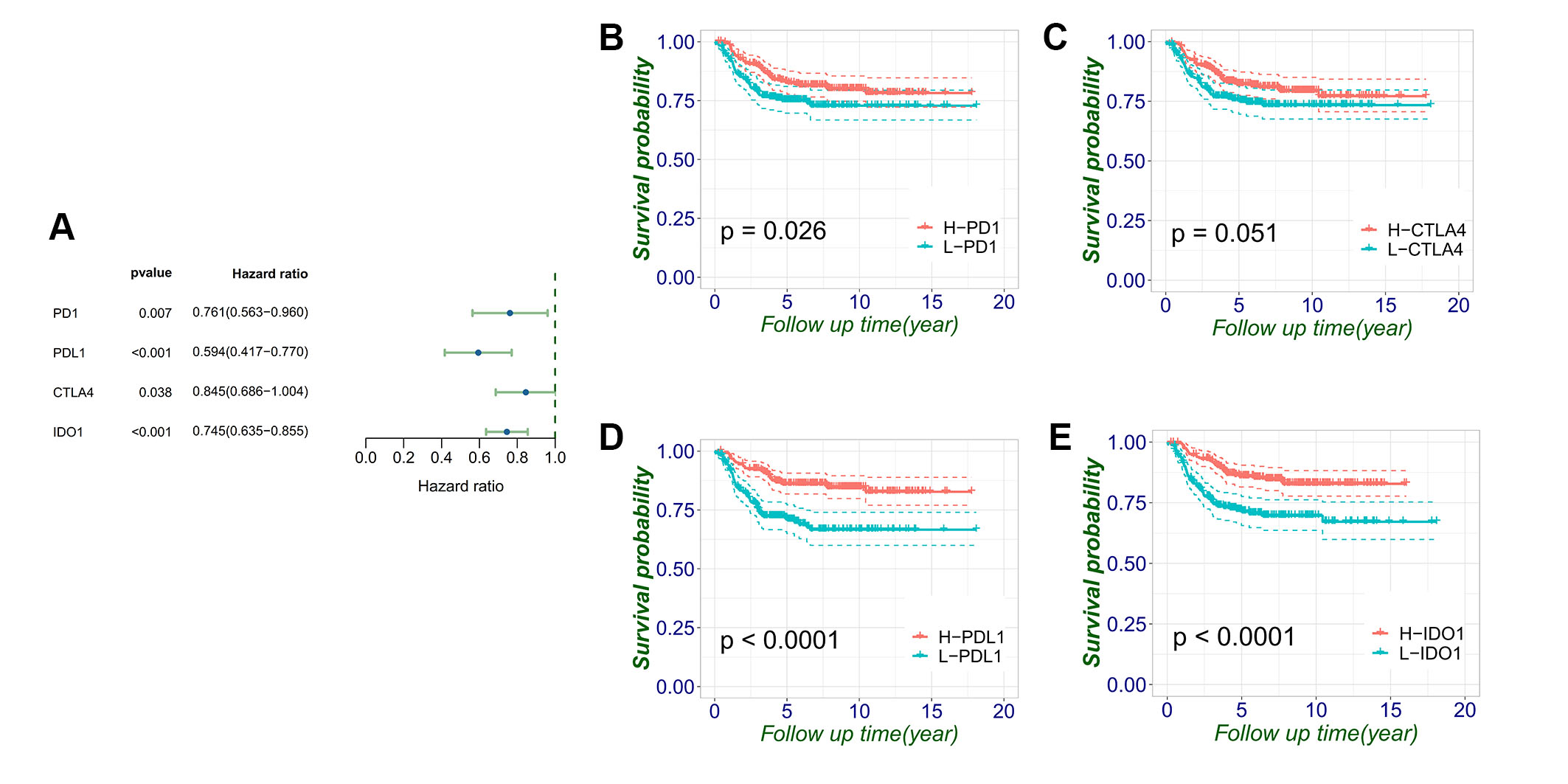

Supplement: Supplementary file 7 [file Image6.JPEG]
